# Young@Heart: empowering the next generation of cardiovascular researchers

**DOI:** 10.1007/s12471-020-01454-6

**Published:** 2020-08-11

**Authors:** M. F. Hoes, A. S. J. M. te Riele, M. M. Gladka, B. D. Westenbrink, G. P. J. van Hout, M. M. G. van den Hoogenhof, A. Ghigo, S. Bollini, N. H. Purcell, S. M. A. Sohaib, I. Kardys, D. W. D. Kuster

**Affiliations:** 1 Young@Heart, the Dutch CardioVascular Alliance, Utrecht, The Netherlands; 2 Department of Cardiology, University Medical Center Groningen, University of Groningen, Groningen, The Netherlands; 3 Department of Cardiology, University Medical Center Utrecht, Utrecht, The Netherlands; 4 Hubrecht Institute, Royal Netherlands Academy of Arts and Science, Utrecht, The Netherlands; 5 European Society of Cardiology, Scientists of Tomorrow, Biot, France; 6 Junior Chamber Board of the Netherlands Society of Cardiology, Utrecht, The Netherlands; 7 Institute of Experimental Cardiology, University of Heidelberg, Heidelberg, Germany; 8 Partner Site Heidelberg/Mannheim, DZHK (German Center for Cardiovascular Research), Heidelberg/Mannheim, Germany; 9 Department of Molecular Biotechnology and Health Sciences, Molecular Biotechnology Center, University of Torino, Torino, Italy; 10 International Society of Heart Research, Durham, NC USA; 11 Department of Experimental Medicine (DIMES), Regenerative Medicine Laboratory, University of Genova, Genova, Italy; 12 Basic Cardiovascular Sciences Early Career Committee, American Heart Association, Dallas, TX USA; 13 Department of Pharmacology, School of Medicine, University of California San Diego, La Jolla, CA USA; 14 European Society of Cardiology Board Committee for Young Cardiovascular Professionals, Biot, France; 15 King George Hospital, Essex, UK; 16 St Bartholomew’s Hospital, London, UK; 17 Department of Cardiology, Erasmus MC, University Medical Center Rotterdam, Rotterdam, The Netherlands; 18 Department of Physiology, Amsterdam Cardiovascular Sciences, Amsterdam UMC, Vrije Universiteit, Amsterdam, The Netherlands

**Keywords:** Early career researchers, Cardiovascular research, Dutch CardioVascular Alliance

## Abstract

In recognition of the increasing health burden of cardiovascular disease, the Dutch CardioVascular Alliance (DCVA) was founded with the ambition to lower the cardiovascular disease burden by 25% in 2030. To achieve this, the DCVA is a platform for all stakeholders in the cardiovascular field to align policies, agendas and research. An important goal of the DCVA is to guide and encourage young researchers at an early stage of their careers in order to help them overcome challenges and reach their full potential. Young@Heart is part of the DCVA that supports the young cardiovascular research community. This article illustrates the challenges and opportunities encountered by young cardiovascular researchers in the Netherlands and highlights Young@Heart’s vision to benefit from these opportunities and optimise collaborations to contribute to lowering the cardiovascular disease burden together as soon as possible.

## Dutch contribution to the field

- Dutch cardiovascular alliance (DCVA) is a platform that combines stakeholders in the cardiovascular field with the goal to lower cardiovascular disease burden by 25% in 2030.
- Young@Heart is part of the talent pillar of DCVA and represents young researchers in the Netherlands.
- Young@Heart aims to provide career perspectives in academic, corporate and non-governmental settings for talented basic and clinical scientists.
- By providing workshops, national and international networking and funding opportunities, Young@Heart hopes to ensure a bright future for cardiovascular research in the Netherlands.

## Introduction

Cardiovascular disease poses a major clinical and economic burden to healthcare systems worldwide, and it is set to be the biggest health risk of the future. This threat is recognised by the government and other major stakeholders in the Netherlands, and large investments are being made to reduce this risk. This article illustrates the way cardiovascular science is governed in the Netherlands, and highlights challenges and opportunities in the future course of cardiovascular research from the perspective of early career scientists.

In the Netherlands, cardiovascular research is mainly funded by the government through the Dutch Research Council (NWO, all disciplines) and the Netherlands Organisation for Health Research and Development (ZonMw), as well as the Dutch Heart Foundation. These parties have formulated priorities in an agenda to direct current and future research. Their support consists of opening various funding opportunities and by hosting courses, workshops and events. Various recurring grants are awarded to support research at levels ranging from individual researchers to multicentre national consortia. To further expand this approach, the Dutch Heart Foundation and NWO/ZonMW, together with the Netherlands Federation of University Medical Centres (NFU) and the Royal Netherlands Academy of Arts and Sciences (KNAW) collectively launched the Netherlands Cardiovascular Research Initiative (CVON) in 2010. This program was aimed at forming multicentre national consortia in a collaborative effort to enable excellent translational research and valorisation within preselected themes over 5 years. The CVON initiative was later expanded with additional partners to officially form the Dutch CardioVascular Alliance (DCVA) in 2018. Today, these partners include all universities, patient organisations, physician associations, and funding partners (https://DCVAlliance.nl/). The DCVA will continue to support ongoing CVON consortia and new DCVA consortia will be launched.

## One alliance to connect all stakeholders

The DCVA was founded as a result of a collaborative effort to combine the strengths of all major stakeholders in the Netherlands with a specific mission: *to lower the cardiovascular disease burden by 25% in 2030*. The partners were brought together to act in unison, align funding and coordinate the selection of research topics that warrant most attention. A notable and timely example of such DCVA endeavours is the rapid launch of the CAPACITY COVID Registry (https://capacity-covid.eu/) in response to the emerging outbreak of COVID-19.

**Fig. 1:**
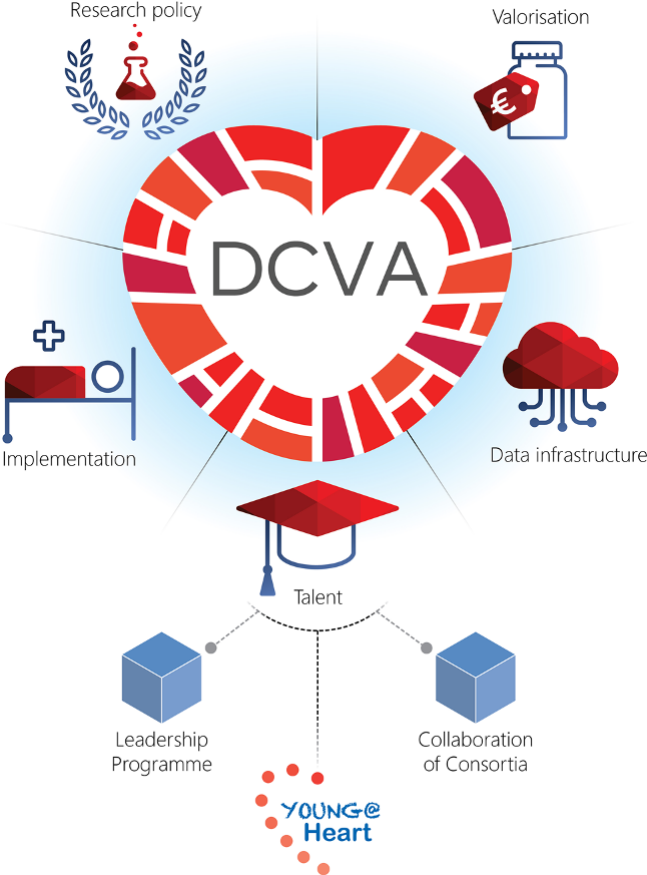
The five pillars of the Dutch CardioVascular Alliance: Research policy, Valorisation, Implementation, Talent, and Data infrastructure. Through these pillars, the DCVA aligns stakeholder efforts to optimise policies and funding opportunities. The talent pillar governs the Leadership Programme, Collaboration of Consortia, and supportsYoung@Heart

The DCVA is divided into five divisions, or *pillars *(Fig. 1). In this article, we will focus on the *talent pillar*. Through the talent pillar, the DCVA guides young researchers in their careers and helps them reach their full potential, which will benefit the cardiovascular field in the future. As part of this endeavour, the Leadership Programme was launched this year to support future leaders in the field, and efforts are being made to facilitate activities for young talent within existing consortia, as well as close collaboration between these consortia. A substantial part of the talent pillar is formed by Young@Heart, a community of early career scientists that represents and supports young researchers, which was an initiative by the Netherlands Heart Institute (previously Interuniversitair Cardiologisch Instituut Nederland [ICIN]) [1].

## Young@Heart: link between the DCVA and young scientists

Young@Heart is an organisation of talented young scientists from the clinical and preclinical arena and is part of the talent pillar of the DCVA. Young@Heart strives to be the voice of the young cardiovascular community. It supports and represents the next generation of scientists and actively reaches out to PhD students, postdoctoral researchers and early career faculty to build a nationwide and diverse community. PhD students are the main focus group of Young@Heart; they are the backbone of cardiovascular research in the Netherlands as the vast majority of this research work is performed by this group.

Young@Heart aims to provide career perspectives in academic, corporate and non-governmental settings for talented basic and clinical scientists. Additionally, it provides ample networking opportunities to support professional and personal development. To that end, Young@Heart hosts biannual events focussed at connecting young researchers with established scientists from academic institutes and prominent corporations. Moreover, connecting young researchers from different institutes will enable them to benefit from each other’s experiences and to form collaborations. Young@Heart also supports the development of grants that are specifically targeted at young researchers. Grants and fellowships have previously been issued to support ambitious, visionary, or even ‘crazy’ (i.e. out-of-the-box) ideas, and Young@Heart coordinates talent grants for PhD students and postdocs with the talent committees of the DCVA consortia. Young@Heart is the first point of contact for young researchers when they have questions, suggestions or needs regarding funding and networking opportunities, policies, and collaboration needs.

Proper representation of the Dutch cardiovascular research community can only be achieved by including talented individuals from different fields and institutes. The goal is to establish a comprehensive network of young researchers from all fields related to cardiovascular research, including clinicians, biomedical and clinical researchers, engineers, and entrepreneurs from all regions of the country. Therefore, Young@Heart emphasises the need to equally represent all individuals involved in cardiovascular research.

**Fig. 2:**
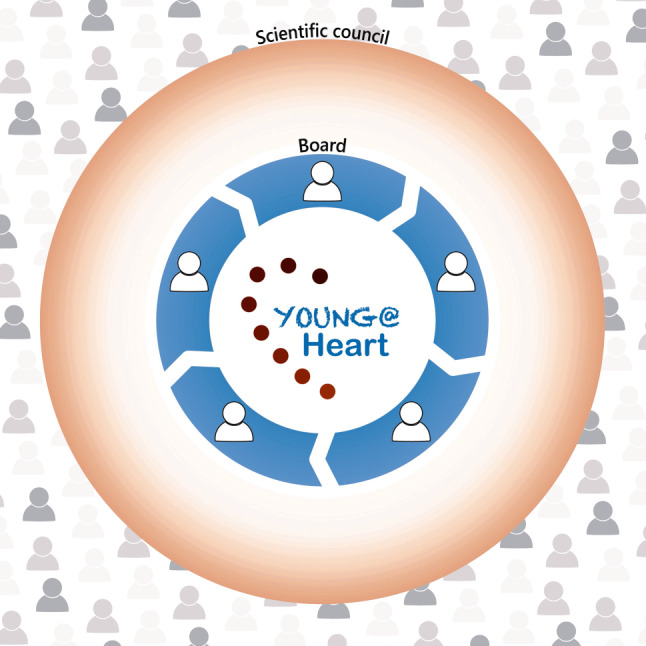
The Young@Heart board consists of five members who are supported by 20–30 scientific council members. Young@Heart aims to be embedded in the young community of cardiovascular researchers

Young@Heart consists of a board of 5 members and a scientific council of 20–30 individuals (Fig. 2). The board consists of scientists or clinician-scientists, of at least postdoctoral level, who have been invited to apply for a board position based on merit and ambition. The board communicates directly with DCVA and the talent coordinators of the DCVA consortia. The scientific council and the board organise regular meetings to discuss policies, upcoming grants, and events. This close relationship between the board and the scientific council inherently results in an extensive network associated with each of its members, which provides an effective platform to connect interested researchers to the DCVA, and vice versa. It is also imperative to Young@Heart to be inclusive and, for instance, equally include women and men in the board and scientific council.

## Challenges for young researchers

PhD students and post-docs who are considering a long-term career in academia often encounter multiple challenges and uncertainties when planning their careers. Should they stay in academia or transfer to a corporate position? Should clinicians specialise while pursuing a scientific career in parallel? Which funding opportunities can they apply for? These are important questions that are often overlooked in the halcyon days at the start of a PhD project.

For researchers choosing to pursue an academic career, obtaining financial support (i.e. grants) is often the first concern in the postdoctoral stage. This is a priority for many, but it applies differently to clinical and basic scientists. Many clinical researchers are medical doctors enrolled in clinical training programmes and their research efforts are often performed in parallel to their clinical obligations, which are almost always urgent and, therefore, prioritised. These clinical duties can be difficult to combine with research-oriented projects and striking the balance between performing experiments (i.e. protected research time) and patient care is the principal challenge in the career path of clinical scientists. To overcome the issue of not being able to fully focus on research, most funding agencies offer specific grants exclusively to clinical researchers or extend the timespan in which grants can be applied for by clinicians.

In contrast, basic scientists have other challenges. Often, they start in a postdoctoral position that is funded by grants obtained by their supervisor. It is paramount for their career that they obtain their own funding. To make the transition to faculty (i.e. assistant professor), it is required to obtain your own funding in the Netherlands. This is especially pressing as non-clinical scientists often have to fund their own salary, whereas salaries of clinical fellows are often paid by their hospitals. Most researchers will apply for the grants listed in Tab. 1. Of course, variations of this theme apply to scientists in different countries. Applying for grants in the Netherlands is perceived to be very competitive and has a quite stringent structure, which benefits people who follow the ideal career track, characterised by a successful PhD that resulted in high-impact publications and an extended time abroad as a postdoc. This system poses considerable challenges for young and motivated researchers and may deter them from embarking on this career path. It is especially challenging during a time in people’s lives when many of them are also starting a family or buying a house. Moreover, there is no guarantee that these curricular prerequisites for successful grant application truly represent the most eligible candidates or scientists.

**Table 1:** Popular grant schemes open to all scientists in the Netherlands

Name	Agency	Size	Duration
*Dekker grants*	*Netherlands Heart Foundation*		
*– Clinical*			
– Junior clinical scientist		€ 165,000	1–2 years
– Clinical scientist		€ 250,000	3 years
– Senior clinical scientist		€ 375,000	2 years (+4 years PhD student, postdoc or technician)
– Clinical established investigator		€ 625,000	5 years
*– Non-clinical*			
– Postdoc		€ 250,000	3 years
– Senior scientist		€ 400,000	2 years (+4 years PhD student, postdoc or technician)
– Established investigator		€ 625,000	5 years
*NWO Talent Scheme*	*Dutch Research Council*		
– VENI		€ 250,000	3 years
– VIDI		€ 800,000	5 years
– VICI		€ 1,500,000	5 years
Clinical fellows grant	ZonMW	€ 200,000	3–6 years

The aforementioned challenges apply to scientists in most fields in the Netherlands, but funding for cardiovascular research is largely channelled into consortia, which can be both beneficial and detrimental for young researchers. Specifically, once a consortium is initiated, members operate within their designated network of collaborators and the need to seek external input or additional collaborators may be diminished. Due to this closed structure, new positions are often filled by researchers from within the consortium. This poses a risk for researchers who are not affiliated with a consortium; increased transparency is necessary to inform these researchers about consortium activities and findings. This is needed to prevent external groups from starting similar projects and to stimulate secondary collaborations as well.

## Opportunities in cardiovascular research

The international cardiovascular research field has seen major and rapid advances. Technological improvements continuously drive increasingly complex study designs. Consequently, agencies invest enormous amounts of time and resources into adjusting and improving their funding policies. The DCVA was founded to align these parties (i.e. the DCVA partners). As part of the DCVA, Young@Heart has a pivotal role in supporting these efforts by bridging the gaps between stakeholders and the next generation of researchers.

Young@Heart recognises the challenges described above, but also considers them to be opportunities. We encourage a synergistic approach for solving these issues. Clinical and non-clinical scientists benefit from one another by offering valuable insights based on their mutual experience. Combining their areas of expertise is crucial in any translational project and should be supported as such. In that respect, consortia work well and provide an invaluable platform for large multicentre projects, but smaller scientific endeavours may be overlooked in such a setting. Thus, there is an urgent need for an extra level of funding directed towards early career scientists. Fortunately, a few calls have recently been opened for research proposals that include two or three applicants per proposal, often with the inclusion of a corporate partner as well (e.g. the Transition Programme for Innovation without the use of animals [TPI]). To further facilitate and encourage this shift in funding opportunities, Young@Heart focuses on raising awareness of all the options medical and biomedical PhD students have; for example, by organising events with speakers from government agencies, corporate partners and various academic institutes (including teachers, biomedical scientists, cardiologists, engineers, etc.). Additionally, Young@Heart encourages young researchers to start forming their own ideas at an early stage and to consciously expand their network in support of those ideas. Consequently, young researchers will be able to make better informed choices and understand the possibilities at an early stage of their careers, or even create possibilities where none existed before. By stimulating early awareness of the funding landscape and early formulation of scientific aims, early career researchers can create new collaborations or join an existing consortium with determination and dedication, which will greatly improve scientific endeavours in general.

In line with this mission, the DCVA aspires to further optimise the way researchers apply for funding. Currently, funding agencies may open calls for applications linked to a specific theme (e.g. innovation to reduce the use of laboratory animals) or calls without a theme. Indeed, the need to balance public interest and successful ongoing research should be met. The DCVA and Young@Heart believe that it is important to be open to researchers with a new brilliant idea, while also recognising the value of very promising results gained from ongoing collaborations and consortia.

## International collaborations

Young researchers are also encouraged to initiate international collaboration, which can be strengthened by applying for collaborative funding. Young@Heart aims to provide access to financial and scientific networks within the Netherlands, but also internationally. One excellent platform is the ERA-Net Cofund action (European Research Area Network co-funded by the European Commission), dedicated to cardiovascular diseases (ERA-CVD). The main goal of ERA-CVD is to facilitate new and existing collaborations across Europe by stimulating international teamwork, to exchange ideas, and to benefit from cross-border expertise. For this purpose, the Joint Translational Call (JTC) was opened annually to promote multidisciplinary translational research projects between researchers from three to five European countries. However, the European Research Council has decided that ERA-CVD will be reformatted from 2021.

Another more accessible way to initiate international collaborations is attending networking events during international conferences. To support these activities, Young@Heart is currently cooperating with other young national and international communities, including the Junior Chamber of the Netherlands Society of Cardiology, the Scientists of Tomorrow (SoT) and Young Communities, which are part of the European Society of Cardiology (ESC), the Early Career Investigator (ECI) sections of the International Society of Heart Research (ISHR), the German Centre for Cardiovascular Research (DZHK), as well as the American Heart Association (AHA). With this initiative, we aim to improve international networking and collaborations; specifically, by organising pre-meetings and sessions tailored to young scientists during major international conferences.

## Conclusion

Scientific output in cardiovascular science of Dutch institutes has been among the best internationally. There is a strong need to maintain and expand top level cardiovascular research in the Netherlands to achieve the goal to substantially decrease the burden of cardiovascular disease. To effectively advance this endeavour, the DCVA brings together all stakeholders in the cardiovascular field in order to align policies and improve collaboration. In this context, Young@Heart represents young researchers and supports their careers, to ensure the future of cardiovascular research in the Netherlands is bright.
